# Epidemiology of *Leptospira* spp. infection in a beef cattle area of Argentina

**DOI:** 10.3389/fvets.2023.1083024

**Published:** 2023-02-21

**Authors:** Mariana Mazzanti, Exequiel Scialfa, Mariana Rivero, Juan Passucci

**Affiliations:** ^1^Centro de Investigación Veterinaria de Tandil (CIVETAN), UNCPBA-CICPBA-CONICET, Tandil, Buenos Aires, Argentina; ^2^CONICET, Buenos Aires, Argentina; ^3^Departamento Zoonosis Rurales Azul, Ministerio de Salud de la Provincia de Buenos Aires, Azul, Buenos Aires, Argentina; ^4^Facultad de Agronomía, Universidad Nacional del Centro de la Provincia de Buenos Aires (UNCPBA), Azul, Buenos Aires, Argentina; ^5^Universidad Nacional del Centro de la Provincia de Buenos Aires, Facultad de Ciencias Veterinarias, SAMP, Tandil, Buenos Aires, Argentina

**Keywords:** *Leptospira*, seroprevalence, risk factors, beef cattle, Argentina, spatial analysis

## Abstract

Leptospirosis is an infectious disease caused by pathogenic *Leptospira* that affect humans and animals. This disease is complex and non-eradicable in nature. Therefore, the understanding of it is epidemiology in different environments is crucial to implement prevention and control measures. The prevalence of *Leptospira* infection in beef cattle farms is affected by multiple environmental, management and individual factors. In this study, a cross-sectional serological survey was carried on to estimate the prevalence of *Leptospira* antibodies in beef cattle in Tandil and Ayacucho Departments (Buenos Aires Province) and to identify risk factors and spatial clusters associated with seropositivity. Using a probabilistic two-stage sampling, 25 farms and 15 animals per farm were selected. The Microagglutination Test was used to analize all serum samples. Bivariate and multivariate analyses were performed. Seventy-three out of 375 cows were seropositive, representing a positivity rate of 19.47% (95% CI: 10.51–28.42), with Sejroe and Pomona being the most reactive serogroups: 9.33% (95% CI: 6.26–12.41) and 8.27% (95% CI: 5.35–11.19), respectively. The prevalence in Ayacucho was 23.11% (95% CI: 10.05–36.17), and in Tandil, 14% (95% CI: 3.25–24.75). The animals from Ayacucho presented 2.01 (1.16–3.49) more chances of being positive compared with those from Tandil (*p* < 0.01). After the Generalized Linear Mixed Model (GLMM) with random effect of farm-level risk, the presence of lagoons (OR: 7.32, 95% CI: 1.68–31.8, *p* < 0.05) and undulating terrain (OR: 0.24, 95% CI: 0.07–0.74, *p* < 0.05) were associated with bovine leptospirosis. Four spatial clusters with higher rates of seropositivity were detected. A new GLMM was performed with the significant variables detected in the first GLMM and a new variable, “being inside the spatial cluster,” being the only one that remained significant (OR: 9.58, 95% CI: 3.39–27.08, *p* < 0.0001). The animals inside the clusters belonged to farms with a greater presence of creeks (OR: 9.03, 95% CI: 3.37–24.18, *p* < 0.0001), higher accumulated rainfall (OR: 1.01, 95% CI: 1–1.01, *p* < 0.0001) and less undulating terrain (OR: 0.18, 95% CI: 0.10–0.35, *p* < 0.0001). We conclude that *Leptospira* is seroprevalent in beef cattle in Tandil and Ayacucho Departments, especially in the latter, where the largest cattle farms are located. Prevalence of seropositivity animals was associated with selected environmental risk factors.

## Introduction

Leptospirosis is a zoonotic disease with a worldwide distribution. It is caused by pathogenic helical spirochetes of the *Leptospira* genus (family *Leptospiraceae*, order *Spirochaetales*), which may affect humans, domestic and wild animals (1). In livestock, leptospirosis can cause economic losses, particularly in developing countries (2). Most infections are caused by either *Leptospira borgpetersenii* or *Leptospira interrogans*. Although clinical signs of the disease caused by these two species are similar, spatial distribution and ways of transmission varies according to the species. *L. interrogans* is commonly acquired from contaminated surface water, while *L. borgpetersenii* is mainly transmitted host-to-host (3).

When an animal that has been infected (at an early age) becomes a carrier, its urine contaminates the moist soil, and foraging areas within the perimeter of the animal. Young, healthy animals of the same species within the same area become infected by the oldest sick animals; the contamitation of surface waters leads to the risk of infection of other animals, whether wild or domestic (4). It is widely known that *Leptospira* can survive for months in the environment under favorable conditions and that alkaline urine in cattle promotes its perpetuation (5). Animals may be maintenance hosts for some serovars but incidental hosts for others. Bovine leptospirosis occurs worldwide and results from infection by several serovars. *Leptospira borgpetersenii* serovar Hardjo (Hardjobovis) is the common strain of this serovar maintained by cattle, but *Leptospira interrogans* serovar Hardjo (Hardjoprajitno) also occurs in cattle in some parts of the world. Pomona, Grippotyphosa and Icterohaemorrhagiae are the serogroups most frequently identified in incidental infections in cattle, and their transmission is related to pigs, rodents and wildlife. The acute and severe forms of leptospirosis (fever, icterus, mortality) are uncommon and frequently associated with sporadic outbreaks in calves caused by incidental serovars. In adult cattle, infection often results in high abortion rates among the infected herds a few weeks after the acute phase of the disease (4). Common signs of leptospirosis include reproductive failure, abortion, stillbirths, fetal mummification, weak calves and agalactia (5). In Argentina, to date, *L. interrogans* serovar Pomona (6) has been isolated as an incidental serovar and *L. borgpetersenii* as a maintenance serovar in Hardjo type Hardjo Bovis (7).

It is important to study the serovars that can infect cattle in a region and to know the ecoepidemiology of the disease for the correct implementation of control and prevention measures in the production system. The prevalence of *Leptospira* is affected by environmental factors and cattle management (8). This study was carried on to estimate the prevalence of *Leptospira* antibodies in beef cattle in Tandil and Ayacucho Departments and to assess the associations between seropositivity and risk factors.

## Materials and methods

### Study area

Ayacucho and Tandil Departments are located in the central-east of Buenos Aires Province, Argentina. This province is part of the Pampas Region of the country, constituting the area with the best conditions for agricultural activities. However, the productive characteristics differ throughout the region. Remarkable climatic, edaphic and physiographic differences have determined various uses and production systems (9). Ayacucho is located within the sub-region called Pampa Deprimida (defined by the Salado river basin) at an average of 74 meters above sea level (37° 09′ S and 58° 28′ W). The climate is temperate, with an average annual temperature of 14.25°C and average rainfall of 870 mm. Livestock, mainly breeding beef cattle, is the mainstay of the economy. Tandil is located in the mountains of the Tandilia system, at an average altitude of 284 meters above sea level (37° 04′ S and 59° 08′ W). The climate is temperate, with an average annual temperature of 13.5°C and average rainfall of 879 mm. The local economy is based on agriculture, livestock and other productions. Ayacucho Department has a cattle population of 683,004 distributed in 1,721 farms, whereas Tandil has 248,696 cattle distributed in 793 farms (10). Ayacucho Department neighbors Tandil. The geographical contiguity within Ayacucho and Tandil districtis leads to flooding occurrence in Ayacucho. This is due to the geological characteristics of Ayacucho district (low altitude and slight slope) and its proximity to the Tandilia System (higher altitude subregion). This system works as a centre of rainwater dispersion toward the Rio de la Plata and the Atlantic Ocean. All the creeks crossing the department, such as Tandileofú, Chelforó, Perdido and Langueyú, have their source in the Tandilia system (9, 11).

### Study design, sampling, and data collection

A cross-sectional serological survey was carried on from September 2017 to December 2020. Using a probabilistic two-stage sampling, 25 farms and fifteen animals per farm were selected. SENASA (Servicio Nacional de Sanidad y Calidad Agroalimentaria) and Fundación Aftosa provided the region's database. In the first stage, the number of farms to be selected was calculated using the ProMesa 1.3 programme (12) using the following formula:


$$\begin{array}{l} n=\frac{p\;x\;\left(1-p\right)\;x\left[ROH\;x\;\left(b-1\right)+1\right]x\;Z^{2}}{e^{2}\;x\;b} \end{array}$$

Where *p* is the expected seroprevalence of cows reactive to *Leptospira, z* is the confidence level, ROH is the homogeneity rate, *b* is the number of animals selected per farm and e is the acceptable (allowable) absolute error. Our assumptions were as follows: a prevalence of 20.33% (13), a relative error of 33%, a low homogeneity rate (0.06–0.12) (14) and 15 animals sampled per farm. The minimum sample size estimated was 25 farms (375 animals). More farms were included from Ayacucho (14) because this department has the largest number of cattle farms. The second stage was conducted on each farm through systematic randomization of the animals. The animals included in the study were bulls and breeding females ≥15 months of age, that were not vaccinated against *Leptospira* spp. or had been vaccinated more than 6 months before sampling. In addition, a questionnaire was administered to collect epidemiological information about the potential risk factors associated with seropositivity to *Leptospira* spp. The survey was based on general production and environmental characteristics, feeding practices, health and reproduction status. All farms were georeferenced using Global Positioning Systems (GPS) to perform spatial analysis and subsequent search for meteorological and satellite information such as the presence of water bodies, potential evapotranspiration, temperature and solar radiation. Water bodies were detected using information from the Landsat 8 satellite (spatial resolution of 30 meters) and calculated with MNDWI (Modified Normalized Difference Water Index) (15) with the QGIS software. The potential evapotranspiration was obtained through the EOS-Terra satellite and estimated using the ENVI software. The temperature was calculated monthly through data from the National Meteorological Service and solar radiation, using information from the sensor CERES (Clouds and the Earth's Radiant Energy System) (16). On the farms, blood samples were collected from the animals by venipuncture. The blood was allowed to clot at room temperature and centrifuged at 1,500 rpm for 15 min. The sera were separated and stored at −20°C.

### Serological testing

The Microagglutination Test (MAT), the reference serological test, was used for processing samples, considering a titer of ≥1:200 as a criterion of positivity. This is the cut-off point used in cattle in Argentina since leptospirosis is an endemic disease and because cattle are vaccinated in areas where this agent is present (17). Antibodies against pathogenic *Leptospira* were detected by the MAT in the Leptospirosis Laboratory, Department of Rural Zoonosis (Ministry of Health of Buenos Aires Province), according to WOAH (18) protocols.

A panel of live antigens of ten *Leptospira* spp. reference strains were used: *L. interrogans* serogroup Canicola serovar Canicola strain H. Utrecht IV, serogroup Hebdomadis serovar Hebdomadis strain Hebdomadis, serogroup Icterohaemorrhagiae serovar Copenhageni strain M20, serogroup Pomona serovar Pomona strain Pomona, serogroup Pyrogenes serovar Pyrogenes strain Salinem, serogroup Sejroe serovar Wolfii strain 3,705 and serogroup Sejroe serovar Hardjo strain Hardjoprajitno, *L. borgpeterseni* serogroup Ballum serovar Castellonis strain Castellon 3 and serogroup Tarassovi serovar Tarassovi strain Perepelitsin and *L. kirschneri* serogroup Grippotyphosa serovar Grippotyphosa strain Castellon 3. This panel was developed at 28–30°C in the Ellinghausen-McCullough-Johnson-Harris (EMJH) medium with no more than 15 days of growth. Serial serum dilutions were performed with phosphate-buffered saline (PBS, pH 7.2) starting from 1:100 dilution. The plates were incubated at 37°C for 90 min. After incubation, the serum-antigen mixtures were checked for agglutination under a dark field microscope. Tests were interpreted as positive when agglutination at ≥ 1:200 of at least 50% of the leptospires for any serogroup was observed. The highest serum dilution with >50% agglutination or ≤50% free leptospires, compared to the negative control, was considered the endpoint titer of quantitative MAT.

### Data analysis

The data about each animal, the characteristics of each farm and laboratory results were entered into an Excel database (Microsoft, Redmond, WA, USA). Prevalence of anti-*Leptospira* spp. antibodies with the 95% Confidence Interval (CI) was estimated. Also, farms positivity was determined. The association between the outcome seropositivity to *Leptospira* spp., to *Leptospira* serogroup Sejroe (adapted serogroup) and to *Leptospira* serogroup Pomona (incidental serogroup) and the variables under analysis was assessed by a bivariate analysis using a Chi-squared test. Fisher's exact test was used if one or more cells expected value was <5. Odds ratios (OR) and 95% CI were also estimated and calculated for each variable. For quantitative variables, parametric or non-parametric tests were used. The null hypothesis was that there were no differences between groups. All the statistical tests were carried out at α=0.05. Quantitative and qualitative variables at *p*-value < 0.2 in the bivariate analysis were analyzed by a Generalized Linear Mixed Model (GLMM) with random effect of farm-level risk. All statistical analyses were performed with software R v. 4.0.2 (10).

Potential spatial clusters were investigated in the study area with space scan statistics using SaTScan software, v10.0.2. Poisson model for high rates was performed for detecting spatial patterns of the number of MAT positive events in a geographical location, taking each farm as a unit for analysis according to a known population at risk (19).

Another qualitative variable called “being inside the spatial clusters” was generated, and an animal was considered to be exposed to the variable when it belonged to a farm inside a significant cluster. The animal was not exposed to the variable when it belonged to a farm outside the significant clusters. GLMM was performed with the significant variables detected in the first GLMM with a new variable, “being inside the spatial clusters.”

### Ethical considerations

This work has been approved by the Ethics Committee according to the Animal Welfare Policy (act 087/02) of the Faculty of Veterinary Medicine (U.N.C.P.B.A, Tandil, Argentina) http://www.vet.unicen.edu.ar.

## Results

**Figure 1:**
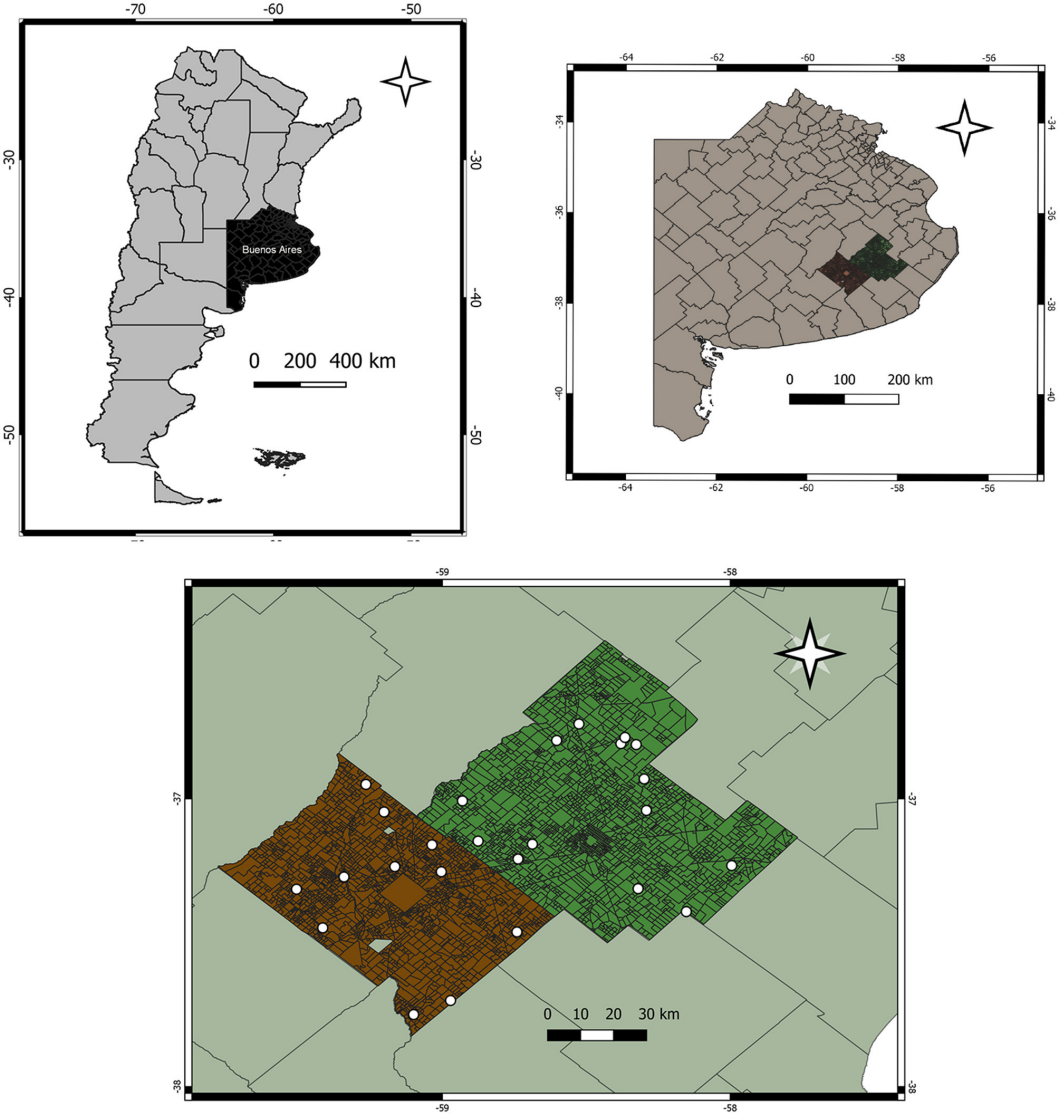
Map of Argentina. Map of Buenos Aires Province. Geographic boundaries of the Departments (Ayacucho and Tandil) and sampling sites.

Twenty-five farms were sampled: 14 from Ayacucho and 11 from Tandil. The spatial distribution of the farms is shown in Figure 1. The size of the farms used for livestock ranged from 45 to 3,500 hectares. The largest farms were those located in Ayacucho. These farms had 100 to 1,800 females' cattle (cows and heifers), and the hectares devoted to livestock varied from 88 to 3,500. In Tandil, the hectares used for livestock ranged from 45 to 770, and the number of females varied from 50 to 550. In both departments, the pregnancy rate varied from 86 to 100% (22 farms), with an average of 92%. The average calving rate was 91% (14 fields), varying from 81 to 100%. These herds had no history of diagnosis of leptospirosis (Figure 1).

Seventy-three out of 375 animals (19.47%; 95% CI: 10.51–28.42) were seropositive, and 72% (95% CI: 52.4–91.6) of the farms had at least one positive animal. Sejroe was the most prevalent serogroup (35/375), 9.33% (95% CI: 6.26–12.41), followed by Pomona (31/375), 8.27% (95% CI: 5.35–11.19), Hebdomadis (30/375), 8% (95% CI: 5.12–10.9), Tarassovi (4/375), 1.1% (95% CI: 0.29–2.71) and Canicola (2/375), 0.53% (95% CI: 0.07–1.91). Cross-reactions occurred between 2 and 3 or more serogroups in 26.03% (19/73) and 6.85% (5/73) of the positive samples, respectively.

Seroprevalence in Ayacucho was 23.11% (95% CI: 10.05–36.17), and in Tandil 14% (95% CI: 3.25–24.75). The animals from Ayacucho presented 2.01 (1.16–3.49) more chances to be positive as those from Tandil (*p* < 0.01). Besides, the farm seroprevalence in Ayacucho 80% (95% CI: 59.05–100.95) was not significaly differente from Tandil 60% (95% CI: 28–92.01(*p* > 0.05).

Tables 1, 2 show epidemiological data, individual factors, structural and management conditions on the farms and other environmental and geographical exposures as well as the relation with the seropositivity to the infection with *Leptospira* spp.

**Table 1:** Qualitative risk factors associated with leptospirosis seropositivity in beef cattle from Tandil and Ayacucho Departments, Buenos Aires Province, Argentina.

**Individual characteristics**					
**Variable**	**Category**	**Positive**	**Negative**	**OR (IC95%)**	***p*** **-value**
Sex	Female	73	291	1	0.1
	Male	0	11	–	
Management factors					
Presence of others species	Goats				
	Yes	8	7	5.19 (1.82–14.81)	<0.001
	No	65	295	1	
	Horses				
	Yes	54	246	0.65 (0.36–1.17)	0.15
	No	19	56	1	
Bovines sharing paddocks with other domestic animals	Yes	18	117	0.52 (0.29–0.92)	<0.05
	No	55	185	1	
	Consociated pastures				
	Yes	52	248	0.54 (0.30–0.97)	<0.05
	No	21	54	1	
	Green grass				
	Yes	11	139	0.21 (0.11–0.41)	<0.001
	No	62	163	1	
Feeding	Natural grass				
	Yes	33	87	2.04 (1.21–3.44)	<0.05
	No	40	215	1	
	Silage				
	Yes	5	115	0.12 (0.05–0.31)	<0.001
	No	68	187	1	
	Stubbles				
	Yes	8	112	0.21 (0.10–0.45)	<0.001
	No	65	190	1	
Alternating types of antiparasitic	Yes	19	160	0.31 (0.18–0.55)	<0.001
	No	54	142	1	
Single use of needle	Yes	35	85	2.35 (1.39–3.97)	<0.05
	No	38	217	1	
Handling and disposal of carcasses	On-site incineration	1	14	1	0.07
	Pit burial without incineration	49	76	3.38 (1.92–5.96)	
	Remains on site of death	23	112	0.37 (0.21–0.65)	
Livestock hectares	45–250	8	82	1	<0.05
	250–475	16	74	0.86 (0.47–1.6)	
	475–964	22	68	1.48 (0.84–2.62)	
	964–3,500	27	78	1.68 (0.98–2.89)	
Total females fit for reproduction	50–400	19	161	1	<0.001
	400–1,800	54	141	3.24 (1.84–5.74)	
Previous diagnosis of infectious diseases causing reproductive losses	Yes	15	30	2.34 (1.19–4.64)	<0.05
	No	58	272	1	
Sampling season	Winter	2	43	1	<0.05
	Autumn	37	143	1.42 (0.68–1.90)	
	Spring	34	86	2.19 (1.30–3.69)	
	Summer	0	30	–	
Environmental factors					
Water bodies	Creeks				
	Yes	17	28	2.97 (1.52–5.75)	<0.001
	No	56	274	1	
	Ditches				
	Yes	17	28	2.97 (1.52–5.75)	<0.001
	No	56	274	1	
	Lagoon				
	Yes	26	34	4.36 (2.39–7.92)	<0.001
	No	47	268	1	
	On the surrounding farms				
	Yes	21	39	2.72 (1.48–5)	<0.001
	No	52	263	1	
	Detected by satellite				
	Yes	54	186	1.77 (1.005–3.14)	<0.05
	No	19	116	1	
Location	Ayacucho	50	160	1	<0.05
	Tandil	23	142	0.51 (0.3–0.89)	
Type of terrain	Depressed soil				
	Yes	59	181	2.81 (1.50–5.27)	<0.001
	No	14	121	1	
	Undulating				
	Yes	16	149	0.29 (0.16–0.52)	<0.001
	No	57	153	1	
	Summer				
	Yes	1	29	0.13 (0.02–0.98)	<0.05
	No	72	273	1	
Floodable in	Autumn				
	Yes	18	132	0.42 (0.24–0.75)	<0.05
	No	55	170	1	
	Winter				
	Yes	61	194	2.83 (1.46–5.49)	<0.05
	No	12	108	1	
Hills on the farm	Yes	3	42	0.25 (0.07–0.83)	<0.05
	No	70	245	1	
Presence of rodents	Yes	65	295	0.19 (0.07–0.55)	<0.001
	No	8	7	1	

Results from bivariate analysis.

**Table 2:** Quantitative risk factors associated with leptospirosis seropositivity in beef cattle from Tandil and Ayacucho Departments, Buenos Aires Province, Argentina.

**Variables**	**Positive (*n* = 73)**	**Negative (*n* = 302)**	**Wilcoxon test, *p*-value**
	**Median (Q1–Q3)**	**Median (Q1–Q3)**	
Cattle density	0.73 (0.53–0.92)	0.83 (0.66–1.88)	<0.05
Medium temperature	13.8 (12.9–16.32)	13.8 (11.1–16.2)	0.74
Solar radiation (clear sky)	197.6 (192.6–355.52)	197.6 (138.44–303.61)	<0.05
Potential evapotranspiration	0.01 (0.0032–0.01)	0.0038 (0.002–0.01)	<0.05
Rainfall accumulated in six months	566.19 (342.79–614.63)	502.41 (473–580.15)	0.08

Results from bivariate analysis.

The GLMM with the smallest Akaike's information criterion (AIC = 310.35) was selected as the best one. The significant predictors that best explained seropositivity to *Leptospira* spp. were the predominance of undulating terrain (OR: 0.24, 95% CI: 0.07–0.74, *p* < 0.05) and presence of lagoons in the fields (OR: 7.32, 95% CI: 1.68–31.81, *p* < 0.05).

**Figure 2:**
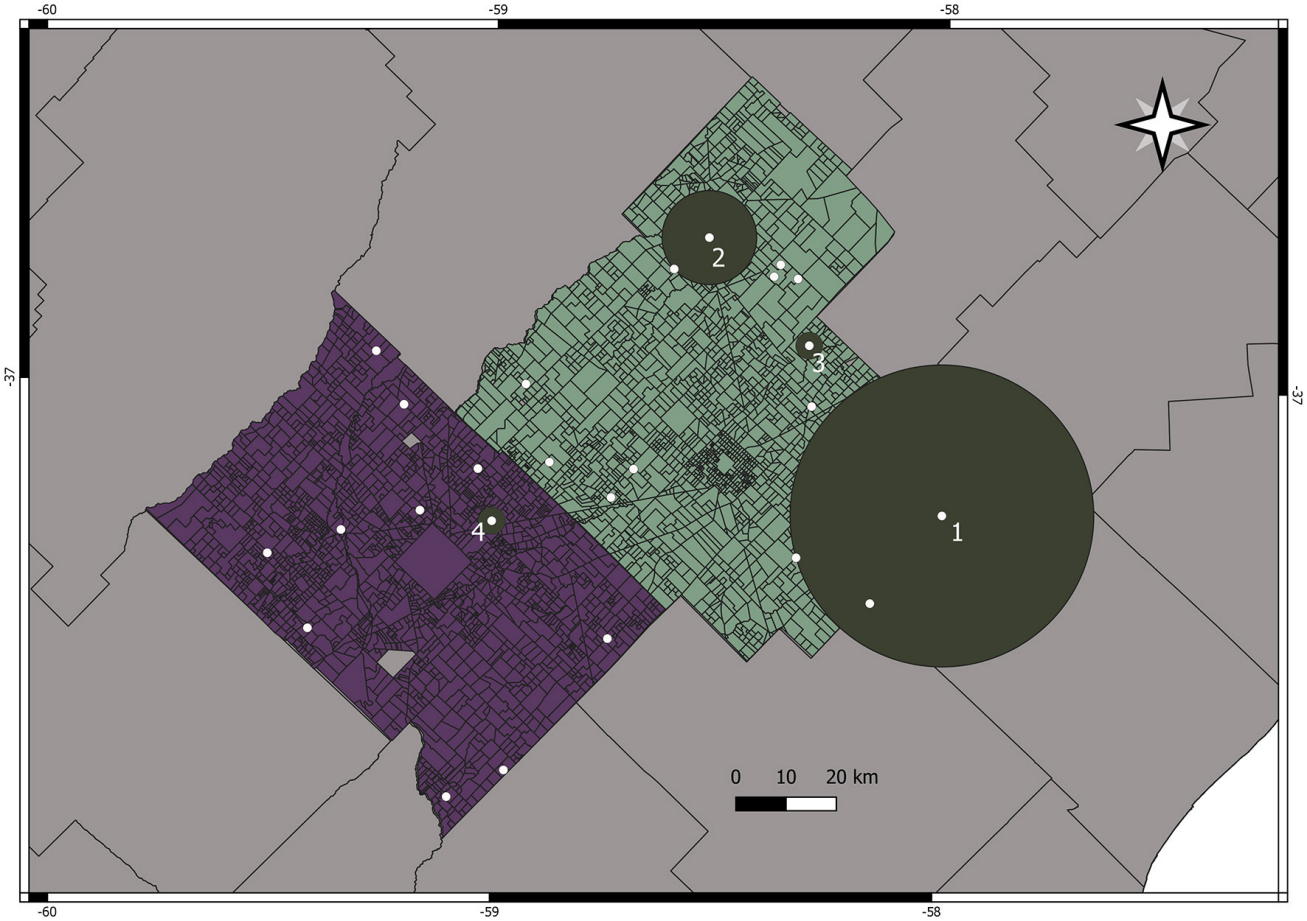
Spatial clusters of higher risk of leptospirosis found in Ayacucho and Tandil Departments.

Concerning the spatial analysis, four high-rate clusters of seropositive cattles were found. Using the Poisson model, a spatial cluster (1) was detected in Ayacucho (37° 13′ 48″ S, 57° 59′ 24″ W; radius: 30.10 km; *p* < 0.001), where the risk of infection was nearly 28 (RR = 28.29) times higher on farms located inside the cluster than on those located elsewhere. A second cluster was detected (2) (36°44′ 24″ S, 58° 31′ 12″ W; radius 9.38 km, *p* < 0.001), where the risk of infection was 8 (RR = 8.47) times higher than on the farms located inside the cluster. Two other clusters (3 and 4) were detected in Ayacucho (36°55′48″ S, 58°17′24″ W; radius 0 km; *p* < 0.001), where the risk of infection was 22 (RR = 22.27) and in Tandil (37° 15′ 12.34″ S, 59° 0′ 13.28″ W, radius: 0 km; *p* < 0.001), where the risk of infection was 9.16 (Figure 2).

When a new GLMM was performed including the significant variables detected in the first GLMM and the new variable “being inside the spatial clusters,” the only variable that remained significant was the new one (OR: 9.58, 95% CI:3.39–27.08, *p* < 0.001) (Table 3). This model was the best Akaike had (AIC: 297.61).

**Table 3:** Leptospirosis seropositivity predictors in beef cattle from Tandil and Ayacucho Departments as determined by the Generalized Linear Mixed Model (GLMM) with random effect of farm-level risk.

**Parameters**	**Coefficient (Coeff.)**	**Standard error (SE)**	**Coeff./SE**	***p*-value**	**OR**	**CI (95%)**
Constant	−2.42	0.39	−6.15	<0.0001		
Lagoons	0.49	0.56	0.87	0.3843	1.63	0.54–4.89
Undulating terrain	−0.41	0.49	−0.84	0.3981	0.66	0.25–1.73
“Being inside the spatial cluster”	2.26	0.53	4.24	<0.0001	9.58	3.39–27.08

Considering the last analysis, factors associated with the variable “being inside the spatial clusters” were assessed through bivariate and multivariate analyses. Results from the bivariate analysis of qualitative and quantitative variables are presented in Tables 4, 5, respectively.

**Table 4:** Qualitative variables associated with the variable “being within the cluster” in beef cattle from Tandil and Ayacucho Departments, Buenos Aires Province, Argentina.

**Variable**	**Category**	**Inside the cluster**	**Outside the cluster**	**OR (CI 95%)**	***p*-value**
Management factors					
	Ditches				
	Yes	30	15	30 (13.33–67.49)	<0.001
	No	15	225	1	
	In the surrounding fields				
Water bodies	Yes	45	15	12.75 (6.66–24.39)	<0.001
	No	60	255	1	
	Lagoon				
	Yes	45	15	12.75 (6.66–24.39)	<0.001
	No	60	225	1	
	Creeks				
	Yes	30	15	6.80 (3.47–13.30)	<0.001
	No	75	255	1	
	Undulating				
Type of terrain	Yes	15	150	0.13 (0.07–0.24)	<0.001
	No	90	120	1	
	Depressed soil				
	Yes	90	150	4.80 (2.64–8.72)	<0.001
	No	15	120	1	

Results from bivariate analysis.

**Table 5:** Quantitative variables associated with the variable “being within the cluster” in beef cattle from Tandil and Ayacucho Departments, Buenos Aires Province, Argentina.

**Variable**	**Positive (*n* = 105)**	**Negative (*n* = 270)**	**Wilcoxon test, *p*-value**
	**Median (Q1–Q3)**	**Median (Q1–Q3)**	
Density	0.55 (0.51–0.92)	0.86 (0.70–1.18)	<0.001
Potential evapotranspiration	0.01 (3.2E-03–0.01)	3.7E-03 (1.9E-03–0.01)	<0.001
Rainfall accumulated in six months	614.63 (540.90–623.98)	484.19 (465.35–566.19)	<0.001
Medium temperature	13.40 (12.90–14.70)	14.85 (11.09–16.32)	0.23
Solar radiation	197.50 (138.44–231.90)	220.79 (137.85–304.64)	0.95

Results from bivariate analysis.

In the logistic regression model, the variables that best explained the variable “being inside the spatial clusters” were the presence of creeks on the farms (OR:9.03, 95% CI:3.37–24.18, *p* < 0.0001), the accumulated rainfall (OR:1.01, 95% CI:1–1.01, *p* < 0.0001), and the predominance of undulating terrain (OR:0.18, 95% CI:0.10–0.35, *p* < 0.0001) (Deviance:290, *p*-value:1 and df: 371).

Finally, a multivariate study was carried out to determine factors associated with the seroprevalence of *Leptospira* serogroup Sejroe and the seroprevalence of *Leptospira* serogroup Pomona. The significant variables found in the previous models and the presence of sheeps, goats and swines and the density of bovines were included in the model. In both cases, the presence of lagoons in the fields were associated with the infection (OR: 4.53, 95% CI: 1.19–17.16) (AIC:218.59) and (OR: 44.7, 95% CI:1.01–1,964) (AIC:146.86), respectively.

## Discussion

Seroprevalence of leptospirosis found in the departments of Tandil and Ayacucho (19.47%) was similar to that reported by Linzzito et al. (20), with 20.33% of serologically positive cattle in the Cuenca del Salado region. However, these results differ from a study carried out in the insular region of the Paraná River Delta (Campana, Buenos Aires Province), where the prevalence found was 33.67% (out of a total of 199 animals studied) (21). The differences could be explained by the edaphoclimatic conditions that are different from those in the present study since insularity characteristics are favoirs the maintenance of *Leptospira* spp. (22). Also, the few records of diagnosis due to losses of *Leptospira* spp. on the farms studied in this research and the high prevalence recorded are evidence that the agent is probably underdiagnosed in our region.

The most reactive serogroups were Sejroe 9.33% (95% CI: 6.26–12.41), Pomona 8.27% (95% CI: 5.35–11.19) and Hebdomadis 8% (95% CI: 5.12–10.9). These results are in agreement with clinical reports associated with abortions, stillbirths and calf mortality in Argentina (6). Recently, episodes of reproductive losses associated with high titres of antibodies have been detected against serovars adapted to cattle, such as L. Hardjo and L. Wolffi (23). The most frequent serovars found by Gamietea et al. (21) were Pomona (32.66%), Wolffi (27.64%), Castellonis serogroup Ballum and Icterohaemorrhagiae. The differences between the two studies (such as the detection of *L. Icterohaemorrhagiae*) may be due to the important role that wildlife, particularly rodents, may be playing in the Paraná River Delta, a habitat of several wild species (21).

From the begining of the initialy, leptospirosis was considered an occupational or environmental disease: “Harvest Fever,” “Cane Cutter's Disease” (24). For this reason, many studies have focused on sources and environmental risk factors to understand its epidemiology (22, 25). Leptospira has been found in water and soil environments in rural and urban areas (26, 27). Several factors are asociated for perpetuation of the bacteria, such as warm temperature, humid environments, neutral or slightly alkaline pH, and the presence of organic material (22, 28). Consequently, surfe of water and animal overcrowding are likely to be important factors associated to leptospirosis outbreaks (27, 28). Likewise, in this work, the models that explained *Leptospira* seropositivity were the presence of lagoons and upper soil environments—probably due to the effect of water drainage—and the being inside the spatial clusters. Furthermore, the spatial clusters were determined by the presence of creeks, millimeters of rainfall accumulated in the last 6 months, and undulating terrain. This was also demonstrated in other studies where leptospirosis notifications were asocciated after rainfall (25, 29). Also, in a previous study, isolated *Leptospira* spp. from soil and water bodies (30). Contaminated water is one of the primary sources of leptospirosis for humans and animals (25, 26, 31). Some reports describe the successful isolation of pathogenic and virulent leptospires from freshwater or soil (31, 32). Also, the survival ability of leptospires for long periods has been reported (31).

The results of this study showed the high presence of the adapted-to-cattle serogroup and the incidental Pomona serogroup. The presence of small ruminants and swines did not explain *Leptospira* seropositivity in either adapted or incidental serogroups. The bovine density was not relevant either to explain the seropositivity to *Leptospira* since these farms continue to be mostly extensive production and do not concentrate many animals per land area. For this reason, it is necessary to consider which wildlife species and niches are found in the area because they are also carriers of several serovars (33, 34).

In the final model, the variable that best explained *Leptospira* seropositivity was to belong to spatial cluster. In other words, the geographic area was associated to high prevalence of infection. The fact that this factor has been found can only make us consider the need for new explorations to determine potential risk factors that are more relevant. Knowledge about environmental factors and determinants for the survival of pathogenic leptospires in the environment remains scarce, and contributes to the inadequate understanding of the basic features of leptospirosis epidemiology (35). More precisely, the association of the survival ability of different strains and the environmental conditions remains largely uninvestigated (22).

The animals in farms located at higher altitudes had a lower risk of infection, probably because, in these sites, the formation of water bodies is less likely, since the water drains down slope. According to Bierque et al. (35), leptospires would be resuspended by rain and existing water particles in the soil, accompanied by the effect of soil washing. Therefore, less positivity will be expected in the high areas because water drains to lower areas.

The spatial clusters found in Ayacucho were expected due to the detection of adverse environmental factors. At the same time, given the better agriculture aptitudes, Tandil has less herds than Ayacucho. Consecutively, there is a management practice that involves sending cows in the last third of pregnancy to pastures with lower yields and forage quality (low soils) so that they do not increase their body condition and thus prevent possible dystocia (36). This must be considered because it can increase the risk of diseases that cause pregnancy loss, so it is imperative to use preventive measures to avoid outbreaks of abortions.

A preventive measure to implement is to vaccinate against *Leptospira* spp. especially when moving animals, in environments where the infection is endemic and the land's characteristics favor the agent's survival. Particularly in Departments such as Ayacucho, where more water bodies are expected to appear due to its territorial conditions that do not favor water drainage. If the animals have not received a vaccination, especially in young categories, heifers or pregnant cows, these animals should not be sent to low pastures where the ground is flooded or has these potential characteristics. Likewise, it is recommended to avoid large extensions of water bodies, such as lagoons, because it is a determining factor for the concentration of fauna that comes to the water supply, in addition to the fact that they are lands less traveled by man. Cows, despite having waters troughs, often use these water sources for consumption and to reduce their body temperature. For this reason, drinkers are suggested to ensure water circulation and reduce the possibility of contamination.

It is worth mentioning that although this study had a two-stage and randomized sampling, only the farms whose owners agreed to participate in the study were accessed, which may have generated a sample selection bias. This could be associated with the fact that they were farms where the workers suspected they had a problem with this disease or it seemed relevant to them to have more knowledge about the health status of their herds.

## Conclusions

Our findings suggest that leptospirosis is endemic in the departments of Tandil and Ayacucho. In addition, the predominance of undulating terrain may decrease the seroprevalence of leptospirosis. Also, the presence of lagoons on the farms would increase the seroprevalence. Ayacucho exhibited a higher seroprevalence of *Leptospira*, which could generate potential productive and reproductive losses. Therefore, it is essential to consider these factors to implement prevention measures that reduce the risk of animal and human infection. The more preventive measures implemented in animals, the lower the risk of exposure to humans (One World, One Health).

## Data availability statement

The original contributions presented in the study are included in the article/supplementary material, further inquiries can be directed to the corresponding author.

## Ethics statement

The study was approved by the Ethical Committee of Animal Welfare (Facultad de Ciencias Veterinarias, UNCPBA) ResCA 087/02.

## Author contributions

Design of the study, critical revisions, supervision, and administration project: JP and MR. Sample collection and writing original draft preparation: MM. Laboratory analysis: ES and MM. Data analysis and interpretation: JP, MM, and MR. Writing of the revised manuscript: MM and MR. All authors have read and agreed to the final published version of the manuscript.
